# Elective ventilation as an improvement measure in organ and tissue donation process

**DOI:** 10.1186/2197-425X-3-S1-A900

**Published:** 2015-10-01

**Authors:** F Segura, D Daga, A Puerto, J Pérez-Vacas, A Fernández-Porcel, MA Frutos

**Affiliations:** Hospital Universitario Virgen de la Victoria, ICU, Málaga, Spain; Transplant Coordination, Sector Málaga, Málaga Spain

## Introduction

Elective Ventilation (EV) is a concept that consists on the agreement reached between patient relatives and transplant coordinator of intubate, connect to mechanical ventilation and admit in intensive care unit those people who are in a coma secondary to a catastrophic cerebral vascular accident (CVA), with the only purpose of becoming organ donors after brain death state has been set.

## Objectives

EV protocol impact assessment in donation and transplant hospital programme.

## Methods

Descriptive retrospective study of a series of patients to whom EV was applied during years 2013-2014 at Hospital Universitario Virgen de la Victoria in Málaga. Before relatives interview, it was made sure that the patient case had been neurosurgically dismissed, that there was not obvious donation contraindication and that, one organ at least, met analytic and ecographic criteria of potential viability. Different criteria were analysed such as age, brain death cause, time to brain death, viable organs extracted, relative weight of EV donors among all the effective donors and relatives refusal percentage. Categoric variables shown as numbers and percentages, quantitative variables in terms of median and standard deviation.

## Results

16 EV candidates were registered. After two relative refusals (12.5%), EV was finally applied to 14 patients. The series age was set in 77.07 ± 9.15 years. 12 of the total CVA (75%) were hemorrhagic. In all cases, brain death was determined after 2.21 ± 2.00 days of admission. Twelve of these patients generated 23 valid organs (85.71%, 1.64 organs per patient), making one cardiac, 10 liver and 12 renal transplants possible, meaning 50% of total cardiac transplants, 45.45% of liver transplants and 24% of renal transplants; and globally 31.08% of the total number of transplants generated in our hospital during the study period.

## Conclusions

Potential donors inclusion in EV protocol has led to a remarkable increase in the number of organs available for transplant in our hospital, with an acceptable number of relatives refusals. Despite the age profile of the patients, 85% of them generated viable organs for transplant.
